# Autosomal recessive hereditary hypotrichosis simplex: A case report

**DOI:** 10.1016/j.jdcr.2024.11.019

**Published:** 2024-11-29

**Authors:** Esraa A. Shaheen, Sahal J. Samarkandy, Renad A. Abbas, Rahaf Fadil Alturkistani, Abdullah Ayman Aman

**Affiliations:** a College of Medicine, King Saud bin Abdulaziz University for Health Sciences, Jeddah, Saudi Arabia; b KAIMRC, King Abdullah International Medical Research Center, Jeddah, Saudi Arabia; c Department of Dermatology, King Abdulaziz Medical City, Jeddah, Saudi Arabia; d Department of Dermatology, King Fahad General Hospital, Jeddah, Saudi Arabia

**Keywords:** autosomal recessive, case report, hereditary hypotrichosis simplex, pediatric

## Introduction

Hereditary hypotrichosis (HS) comprises a large and heterogeneous group of disorders characterized by a paucity of hair since birth. These diseases can be classified according to their mode of inheritance and the presence of extracutaneous features such as cardiomyopathy or lymphedema. A rare form of autosomal recessive HS was identified in 2017, caused by mutations in the lanosterol synthase (2,3-oxidosqualene-lanosterol cyclase [LSS]) gene,^1^ which encodes a key enzyme in the cholesterol biosynthetic pathway.^2^ We report the first case of a pediatric patient in Saudi Arabia presenting with diffuse and progressive scalp hair loss due to an LSS gene mutation.

## Case report

A 21-month-old Saudi Arabian boy presented to our dermatology clinic with sparse, thin, and short hair, which did not grow quickly and was easily lost since birth. His eyelashes and eyebrows were unaffected. The patient did not exhibit any other features, such as palmoplantar keratoderma or dysplasia of the nails and teeth. His medical history lacked any disorders, including developmental delay. There was a positive history of consanguinity (third-degree relative) between the parents. Moreover, he has a positive family history of 2 cousins who have sparse hair since birth.

**Fig 1:**
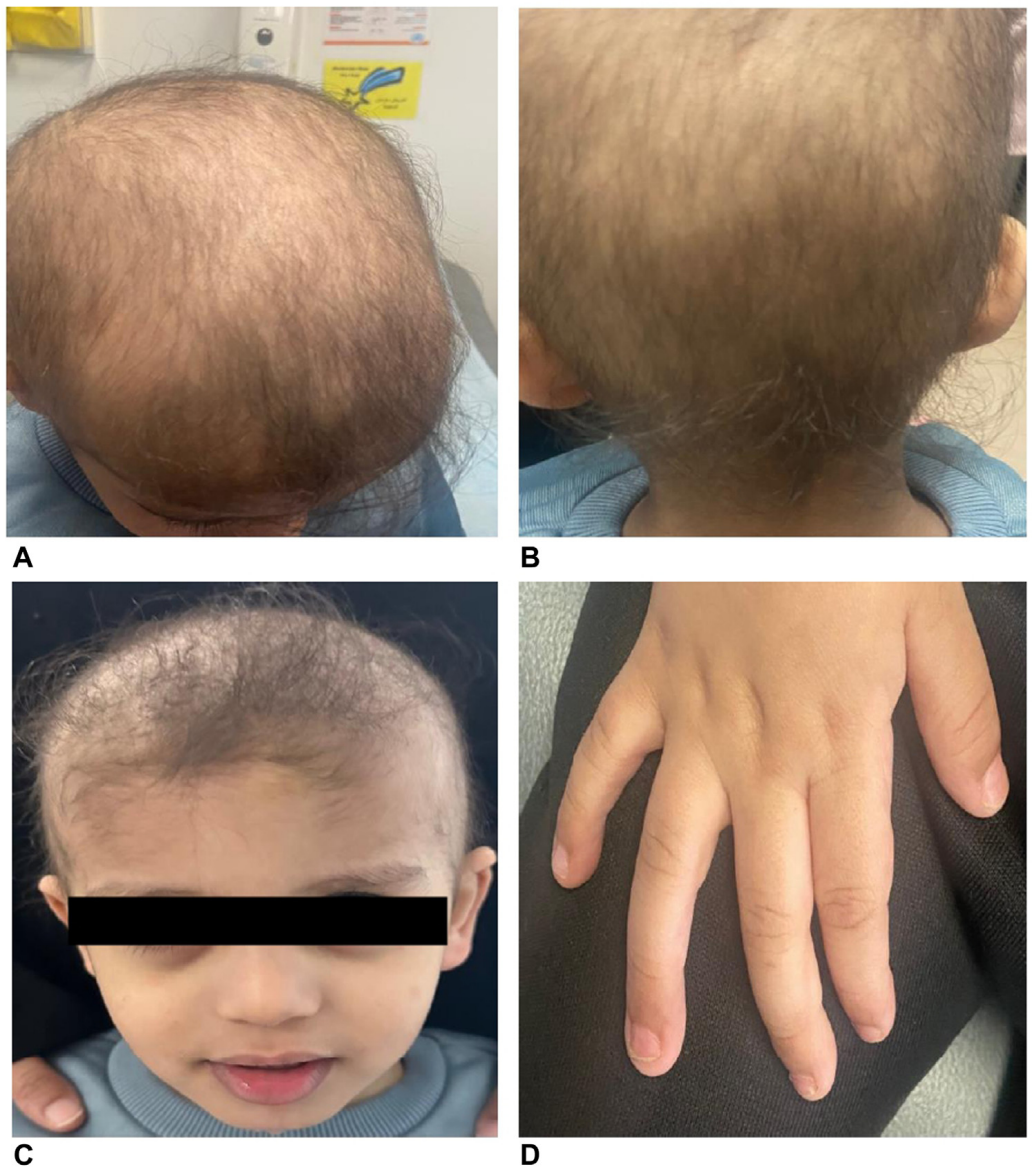
Illustrates the clinical presentation of the patient, showcasing: (**A** and **B**) diffuse hair thinning, (**C**) sparse eyelashes and eyebrows, and (**D**) absence of nail dystrophy.

Physical examination revealed diffuse thinning of the hair, as observed by trichoscopy. No hair shaft abnormalities were noted, and the scalp vellus hair was intact (Fig 1, *A*-*C*). The patient did not have dysmorphic features, teeth abnormalities, or nail findings (Fig 1, *D*).

Whole-exome sequencing TRIO (typically refers to a group of three individuals, often comprising the parents and their child, whose genetic data is studied) identified a homozygous variant of uncertain significance (VUS) in the LSS gene, with both parents being heterozygous carriers. This variant is associated with hypotrichosis-14.

Following the confirmation of autosomal recessive hereditary hypotrichosis simplex, management focused on counseling the patient’s family about realistic expectations and available treatment options. The use of topical minoxidil was suggested to possibly enhance hair appearance. Genetic counseling was provided to discuss inheritance patterns and implications for future generations. Regular follow-up visits were scheduled to monitor treatment response and provide ongoing support.

## Discussion

Hypotrichosis is a heterogeneous group of disorders characterized by sparse or absent hair caused by gene mutations.^1^ In this patient, a homozygous variant of VUS in the LSS gene was identified and hypotrichosis-14 was diagnosed. Hypotrichosis-14 is a rare form of autosomal recessive nonsyndromic hypotrichosis marked by sparse or absent lanugo-like hair on the scalp, as well as reduced or missing eyebrows, eyelashes, and body hair due to mutation of LSS gene.^3^ LSS is a key enzyme in the cholesterol biosynthesis pathway, catalyzing the first step in the biosynthesis of cholesterol, steroid hormones, and vitamin D.^4^ The molecular mechanisms of LSS mutations related to hair loss are not exactly clear, as mutations in the LSS gene have been associated with variable phenotypes including HS, severe neuroectodermal syndrome with HS, and congenital cataracts.^5^ However, there is an explanation for the mutations in the LSS gene. It disrupts this enzymatic function, leading to altered sterol metabolism and cellular homeostasis. Specifically, deficiencies in LSS activity can impair the production of lanosterol, a precursor for cholesterol synthesis. Cholesterol is vital for maintaining cell membrane integrity and is also involved in the formation and function of lipid rafts, which are essential for various cellular processes including cell signaling and differentiation. In the context of hair follicle biology, the synthesis and regulation of cholesterol and its derivatives are fundamental for normal hair growth. Consequently, mutations in LSS that lead to reduced cholesterol synthesis can result in defective hair follicle development and maintenance.^2^^,^^5^

Previous case reports on LSS gene mutations have been identified in several populations, including Chinese,^4^^,^^6^ German,^2^ Japanese,^7^ Iraqi, Syrian, Georgian, and Afghan^8^ patients, resembling our case. Additionally, mutations in LPAR6, LIPH, and DSG4 genes have been identified in patients with autosomal recessive hypotrichosis with similar presentations.^1^ For instance, a mutation in exon 6 of the lipase H gene led to HS in a 3-year-old female, who initially had normal hair at birth but developed severe hair loss within the first 6 months, with some improvement by age 3.^9^

Clinically, understanding the genetic basis of hypotrichosis allows for precise diagnosis, genetic counseling, and potentially targeted therapeutic interventions. This case highlights the importance of incorporating genomic sequencing techniques in dermatological practice to unravel the genetic etiology of rare hair disorders, facilitating personalized management strategies. The VUS identified through whole-exome sequencing implies that the precise effect of the mutation on LSS activity remains uncertain, potentially influencing the effectiveness of treatments. While topical minoxidil may offer some improvement in hair density, its benefits in genetic hypotrichosis can be limited. The long-term outcomes of our patient will also involve addressing the psychosocial impact of hair loss, with ongoing support and counseling for both the patient and his family. Genetic counseling will play a crucial role in explaining inheritance patterns and preparing the family for future considerations, including the implications for future children. Regular follow-up will be essential to monitor treatment efficacy and provide continuous support as the child grows. The case of this 21-month-old Saudi Arabian boy with hypotrichosis-14 underscores the significance of genetic analysis in elucidating rare dermatological conditions and emphasizes the need for continued research to enhance our understanding of genotype-phenotype correlations and therapeutic avenues for such disorders.

## Conclusion

Autosomal recessive hereditary hypotrichosis simplex is a rare genetic disorder with a significant impact on the quality of life of affected individuals. Early recognition, accurate diagnosis, and comprehensive management are essential for providing appropriate support and counseling to patients and their families.

## Conflicts of interest

None disclosed.
